# In Vitro Assessment of Apoptotic and Cell Cycle Arrest Analysis on HepG2 Cells by Polyethylene Glycol–Coupled Selenium Nanocomposite Fabricated From *Cassia fistula* Flowers

**DOI:** 10.1155/bmri/6212199

**Published:** 2025-08-09

**Authors:** Senthamaraikannan Yasodha, Sundaram Vickram, Shanmugam Rajeshkumar, Hitesh Chopra, Tabarak Malik

**Affiliations:** ^1^Department of Biotechnology, Saveetha School of Engineering, Saveetha Institute of Medical and Technical Sciences, Chennai, Tamil Nadu, India; ^2^Nanobiomedicine Lab, Centre for Global Health Research, Saveetha Medical College and Hospital, Saveetha Institute of Medical and Technical Sciences, Chennai, Tamil Nadu, India; ^3^Centre for Research Impact & Outcome, Chitkara College of Pharmacy, Chitkara University, Rajpura, Punjab, India; ^4^Department of Biomedical Sciences, Institute of Health, Jimma University, Jimma, Ethiopia; ^5^Division of Research and Development, Lovely Professional University, Phagwara, Punjab, India

**Keywords:** apoptosis, *Cassia fistula*, HepG2, nanocomposite, polyethylene glycol, selenium

## Abstract

Polymer-encapsulated nanocomposite has been proven to have ameliorative effects in the treatment of cancer. The focused objective of the present research is to fabricate polyethylene glycol (PEG)–based *Cassia fistula* flower selenium nanocomposite (CFF-SeNC). The fabricated CFF-SeNC was characterised using a UV-visible spectrophotometer, Fourier transform infrared spectroscopy (FTIR), scanning electron microscopy (SEM)–energy dispersive x-ray (EDX), and x-ray diffractometer (XRD) analysis. The highest UV-visible absorption spectra were at 355 nm. The oval-shaped, less agglomerated nanocomposite with a rough surface was seen in the SEM results with the elemental composition of carbon, oxygen, and selenium. The XRD analysis results showed the crystalline form of CFF-SeNC. The potency of CFF-SeNC against pathogenic bacteria and fungi proved that CFF-SeNC is effective against the tested fungi *Candida albicans*. The antioxidative assays—DPPH, ABTS, and H_2_O_2_ of CFF-SeNC—revealed the inhibition percent of 85.51% ± 0.79%, 90.53% ± 0.90%, and 84.87% ± 0.80% at maximum concentration (50 *μ*g/mL), respectively. The anti-inflammatory efficiency of CFF-SeNC was found to be 80.61% ± 0.87% and 87.78% ± 0.88% by egg albumin denaturation and HRBCs (human red blood cells) membrane stabilisation assay. The biocompatible nature of CFF-SeNC was tested by brine shrimp lethality activity, which proved that at 48 h, the minimal dose was not toxic; however, mild toxicity was shown at a higher dosage. The anticancer studies of CFF-SeNC in HepG2 cells were active in inhibiting 50% of cells (IC_50_ value) at 27.30 *μ*g/mL. HepG2 cells depicted predominant morphological changes upon treatment with CFF-SeNC, whereas there was no alteration in *Vero* cells. The elaborative study on determining the phases of cell apoptosis was further analysed by fluorescence microscopy, cell cycle arrest, and Annexin V/propidium iodide staining through flow cytometric analysis. The outcome of current research has corroborated the enhanced therapeutical efficiency of PEG-encapsulated selenium nanocomposite with emphasis on the apoptotic effect in hepatocellular carcinoma (HepG2 cells).

## 1. Introduction

*Cassia fistula*, also known as golden shower, is a native plant grown in India. Almost all parts of the plant help treat many lethal diseases, and the plant possesses antiparasitic, laxative, hypoglycemic, antitumor, anti-infertility, antifungal, and antioxidant properties [1]. The plant's flowers treat fever, skin infections, abdominal pain, and leprosy. Like other parts, the flowers are also known for their antibacterial, antifungal, antioxidant, and hypoglycemic activities. Besides the medicinal applications of *C. fistula*, the alkaloids, phenols, and flavonoids [2] present in the plant help in the accumulation of metallic atoms; they are paving the way for synthesising metallic nanoparticles (NPs). Further, synthesising NP from plants has recently gained attention as they help maintain environmental sustainability, minimising several toxic effects [3].

Selenium (Se) is one such metallic element that is vital in regulating the defensive mechanism. More importantly, Se is a prime part of synthesising thyroid hormone [4]. Also, it aids in muscular movement and maintains the overall immune system against any infections. Apart from the above applications, SeNPs are used for cancer treatment. It was noted that an optimum intake of SeNP prevents the risks of cancer [5].

Particularly, Se exhibits excellent results with respect to its activity against liver cancer. Both in vitro and in vivo studies have been carried out using SeNP to understand their hepatoprotective tendency [6]. Coating of polymers on the surface of NPs enhances their biomedical properties. For instance, Adagen was the first PEGylated protein to be approved by the Food and Drug Administration (FDA) to treat immune-related disorders, after which a total of eight polyethylene glycol (PEG)–conjugated proteins were approved for treating various degenerative disorders [7]. The investigation leads to conjugating PEG with NP in order to improve their therapeutic properties. Gold NPs were coated with PEG and used as drug carriers to treat cancer-related diseases. When gold NPs were capped with PEG, cells' transfection efficacy and uptake were increased by 45% [8]. Despite the fact that PEGylated NPs have several advantages towards cancer treatment, limited research has been conducted concerning the conjugation of plant-based SeNPs with PEG.

According to our knowledge, no previous work has studied the design of Se nanocomposite from the flowers of *C. fistula*. Therefore, this study is aimed at fabricating PEG-encapsulated Se nanomaterial from flowers of *C. fistula* to improve the pharmaceutical potency. The fabricated nanomaterial is characterised by ultraviolet-visible spectroscopy, x-ray diffraction, Fourier transform infrared spectroscopy (FTIR), and scanning electron microscopy (SEM). In addition, the anticancer properties of the NPs will be evaluated with a special focus towards the treatment of liver cancer.

## 2. Materials and Methods

### 2.1. Reagents

Sodium selenite (80451), PEG (79133), 2,2-diphenyl-1-picrylhydrazyl (DPPH) (29128), potassium persulfate (52254), and 2,2-azinobis (3-ethylbenzothiazoline-6-sulfonic acid (ABTS) (40157) were purchased from Sisco Research Laboratories (SRL) Chemicals. Dimethyl sulphoxide (DMSO) (102952) and hydrogen peroxide (107209) were purchased from Merck. Rose Bengal agar (M842) and Mueller–Hinton agar (M173) were procured from HiMedia. Ascorbic acid (A7506), diclofenac sodium (D6899), propidium iodide (PI) (P4170), acridine orange (AO) (318337), and Annexin V-FITC were procured from Sigma-Aldrich. Dulbecco's modified Eagle medium (DMEM) (11995065) and fetal bovine serum (FBS) (A5670801) were obtained from Gibco. (3-(4,5-Dimethylthiazol-2-yl)-2,5-diphenyltetrazolium bromide)–MTT (M6494) was purchased from Invitrogen.

### 2.2. Fabrication of *Cassia fistula* Flower Selenium Nanocomposite (CFF-SeNC)

*Cassia fistula* flowers (CFFs) were collected from the local area in Chennai, cleaned, shade dried, and grounded into powder. Two grams of flower powder was added to distilled H_2_O and heated for 15–20 min, which was filtered and used for further research. Ten milliliters of *C. fistula* extract was mixed drop-by-drop with a constant stirring of 90 mL sodium selenite solution (30 mM). This was then ultrasonicated at 50°C for 30 min left overnight in the shaker at RT. The obtained SeNPs were encapsulated in PEG. Encapsulation was done with minor modifications to the methodology reported earlier. Ten milliliters of PEG was added drop-by-drop to 100 mL of SeNP with constant stirring at an appropriate temperature. The resulting CFF-SeNC was dried in an oven for approximately 10 h at a relatively low temperature of 40°C, collected, and analysed for its biopotential [9, 10].

### 2.3. Characterisation Studies of CFF-SeNC

UV-visible spectroscopy (ESICO, Model No. 3375) determined the maximum absorption peak of CFF-SeNC in the wavelength range of 250–650 nm. Functional groups were examined by FTIR (Bruker), ranging from 400 to 4000 cm^−1^. SEM (JEOL JSM IT-800) revealed the size along with their elemental content through energy dispersive x-ray (EDX) analysis. The crystallinity of CFF-SeNC was examined by a D8 Advance x-ray diffractometer (Bruker, Germany).

### 2.4. Antimicrobial Activity of CFF-SeNC

The pathogens *Escherichia coli*, *Pseudomonas aeruginosa*, *Staphylococcus aureus*, *Enterococcus faecalis*, and *C. albicans* were used for studying antimicrobial properties by agar well diffusion method [11]. The above pathogens were procured from the Department of Microbiology, Saveetha Medical College, Saveetha Institute of Medical and Technical Sciences, Thandalam, Chennai. CFF-SeNC was dissolved using distilled water to perform the antimicrobial assay. Each well was loaded with CFF-SeNC (25 *μ*g/mL, 50 *μ*g/mL, and 100 *μ*g/mL) for positive control SeNPs (25 *μ*g/mL) accordingly. Later, incubated overnight at 37°C, the inhibition zone diameter was noted to evaluate the pathogens' sensitivity to CFF-SeNC.

### 2.5. Evaluation of Biopotency of CFF-SeNC: Antioxidative Efficacy

#### 2.5.1. DPPH Activity

The free radical scavenging capacity of CFF-SeNC was estimated using DPPH activity, a slightly changed procedure from previous methods [12]. One milliliter of DPPH reagent (20 *μ*M) was prepared freshly and mixed with sample volumes (10–50 *μ*g/mL) of CFF-SeNC. The mixture was incubated for 30 min in the dark at RT. UV-visible spectroscopy was used to note absorbance at 517 nm.

#### 2.5.2. ABTS Assay

ABTS scavenging assay was carried out with a few modifications in the earlier methodology [13]. ABTS (7 mM) was mixed with potassium persulfate (2.45 mM) and incubated for around 12–16 h in the dark at RT. The absorbance of this solution was adjusted to 0.7 at 734 nm. One hundred and eighty microliters of prepared ABTS reagent was added to 10–50 *μ*g/mL of CFF-SeNC. A 96-well microplate reader was used to analyse the samples after 6 min, along with the standard (ascorbic acid).

#### 2.5.3. Hydrogen Peroxide Scavenging Assay

The H_2_O_2_ activity was determined using the earlier method with minor modifications [14]. The 10–50 *μ*g/mL aliquots of CFF-SeNC were taken, and the solution volume was balanced to 100 *μ*L with phosphate buffer saline (PBS) (50 mM) (pH 7.4). Nine hundred microliters of H_2_O_2_ (2 mM) was added, vortexed, left for 10 min, and quantified at 230 nm.

### 2.6. Anti-Inflammatory Efficacy

#### 2.6.1. Egg Albumin Denaturation Assay

The anti-inflammation capacity of CFF-SeNC was analysed by egg albumin denaturation assay [15]. Fresh egg albumin (0.2 mL) was homogenised with PBS (2.8 mL). Aliquots of CFF-SeNC (10–50 *μ*g/mL) and positive control (diclofenac sodium) were mixed with the albumin solution and kept initially at 37°C for 15 min and later at 70°C for 5 min in the water bath. The absorbance of the reactant solution was noted at 660 nm.

#### 2.6.2. Human RBC: Membrane Stabilisation Assay

The capability of CFF-SeNC to suppress cell damage by protecting the membrane is evaluated by HRBC activity [16, 17]. The fresh blood sample was collected in a sterile tube containing an anticoagulant from the individual. This was centrifuged at 3000 × g, 5 min at room temperature, and HRBC was collected and washed thrice using normal saline. To this, PBS (10 mM, pH 7.4) was added to get at 10% HRBC suspension. Aliquots of CFF-SeNC 10–50 *μ*g/mL, 500 *μ*L PBS, hyposaline (2 mL), and HRBC were added to centrifuge tubes, vortexed gently, and incubated for half an hour at 37°C. The absorbance of the supernatant at 560 nm was measured after centrifuging for 3 min at 3000 × g.

The following formula was used for determining the percentage inhibition of antioxidative and anti-inflammation assays.

 $$\begin{array}{c} \text{Scavenging }\left(\%\right)=\left[\frac{A\text{ control}-A\text{ sample}}{A\text{ control}}\right]*100, \end{array}$$

where *A* *control* is the absorbance of the control, *A* sample is the absorbance of the sample, and Standard is the ascorbic acid (1 mg/mL).

### 2.7. Biocompatibility of CFF-SeNC: Brine Shrimp Nauplius Lethality Assay

CFF-SeNC's biocompatible nature was assessed by the brine shrimp lethality assay with slight modifications in earlier methods [18, 19].

Twenty-eight grams of NaCl was dissolved in 3 L of distilled H_2_O, which was maintained in a specific rectangular container, and brine shrimp cysts were added. This was kept at controlled conditions of temperature 22°C–29°C, sufficient air supply, and a light source for 1–2 days. The hatched nauplii were motile. These nauplii were collected gently and analysed. Saline solution was prepared freshly; 2 mL was added to each well of the 6 well plates. Ten numbers of hatched nauplii were maintained in each well with different concentrations of CFF-SeNC. One control well without a sample was maintained. This was analysed at the end of 24 and 48 h, and the number of live and dead nauplii was noted down.

The rate of viability was calculated using the following formula:

 $$\begin{array}{c} \text{Viability percent}=\left[\frac{\text{number of live nauplii}}{\text{total number of nauplii}}\right]*100. \end{array}$$

### 2.8. Evaluation of Toxicity and Anticancerous Potency

#### 2.8.1. MTT Assay

*Vero* cell line (African green monkey kidney normal epithelial cells) and HepG2 (hepatocellular carcinoma epithelial cells) were procured from the National Centre for Cell Science (NCCS), Pune, India. Cell lines were kept in an incubator at humidity (95%), 37°C, and CO_2_ (5%); the media used for the growth of the cell line was DMEM containing 10% heat-inactivated FBS, 100 U/mL penicillin, and 100 *μ*g/mL streptomycin.

The toxicity and anticancer potency were assessed in *Vero* and HepG2 cell lines through 3-(4,5-dimethylthiazol-2-yl)-2,5-diphenyltetrazolium bromide (MTT) assay [20, 21]. Cells (10^6^ cells/well) were subcultured in sterile 96-well microplates, incubated at 37°C with 5% CO_2_ until the cells grew to the logarithmic stage and reached 80% density. Cells were observed for any dead cells during the growth phase. Then, the media was discarded, and sample CFF-SeNC (10–50 *μ*g/mL) and negative control (sterile molecular grade water) were added in respective wells and left for incubation in a CO_2_ incubator for 24 h. The negative control and treated wells in *Vero* and HepG2 cells were observed for structural changes using a digital inverted microscope (magnification, 20×). Later, cells were washed using PBS (pH 7.4). To each well, MTT (20 *μ*L) from the stock solution (5 mg MTT/mL PBS) was added and kept for 2 h under dark conditions at 37°C for the formation of formazan crystals. DMSO 100 *μ*L was added to each well, which dissolves the formed crystals; absorbance was noted by the microplate reader. The rate of cell viability was calculated using the formula.

 $$\begin{array}{c} \text{Rate of cell viability}=\left[\frac{\text{sample absorbance}}{\text{control absorbance }}\right]*100. \end{array}$$

#### 2.8.2. AO/PI Fluorescence Staining: Apoptotic Study

Apoptosis of HepG2 cells by CFF-SeNC was studied using AO/PI staining [22]. The cells were seeded in a 6-well microplate and kept at 37°C with 5% CO_2_ until it reached 80%–90% confluency. The cells were treated with an IC_50_ dosage of CFF-SeNC for 24 h. This was observed under a microscope, washed with PBS, and stained with AO (10 *μ*L–100 *μ*g/mL) alone, PI alone (10 *μ*L–100 *μ*g/mL), and AO:PI (1:1 ratio) together under dark conditions at RT. After 15 min, the cells were viewed and photographed using FLoid cell imaging fluorescent microscopy.

#### 2.8.3. Cell Cycle Analysis

The phases of the cell cycle were analysed by culturing HepG2 cells in a T25 tissue culture flask with a cell density of 1 × 10^6^ in DMEM serum media. After the cells reached the appropriate confluence, the CFF-SeNC at IC_50_ concentration was used to treat the cells and incubated for 24 h in a 5% CO_2_ incubator at 37°C. Later, the cells were trypsinised, centrifuged, and washed with prechilled PBS. The fixation was done using 70% ethanol at 4°C overnight. The cell pellet was resuspended in PBS (0.5 mL), and 50 *μ*g/mL RNase A (2 *μ*L) and 100 *μ*g/mL PI (50 *μ*L) were mixed and incubated for half an hour in dark condition. The cells in each phase of the cell cycle were determined using a Becton Dickinson Fluorescence-Activated Cell Sorting Lyric flow cytometer (BD FACSLyric flow cytometer) [22, 23].

#### 2.8.4. Annexin V/PI Staining

The stages of apoptotic cells were determined by the Annexin V-PI staining assay. HepG2 cells with a maximum density of 2–4 × 10^6^ cells/200 *μ*L volume of media were maintained and dosed using an IC_50_ concentration of CFF-SeNC. This was pelletised, washed two to three times using cold PBS, and suspended in Annexin V 1X binding buffer. Later, the suspended pellet with cell concentration (10^6^ cells/mL) was stained by Annexin V (5 *μ*L) and PI (5 *μ*L) and incubated for 30 min in dark conditions, and to this binding, buffer (400 *μ*L) was added. The stained tubes were analysed by BD FACSLyric flow cytometer [23].

### 2.9. Statistical Analysis

The results of the experiments performed in triplicate (*n* = 3) are represented as mean ± standard deviation. The results were analysed using GraphPad Prism 5.0 using two-way ANOVA and Bonferroni posttest. The results are considered significant, denoted by “∗” when *p* < 0.001.

## 3. Results

### 3.1. Synthesis of CFF-SeNC

**Figure 1:**
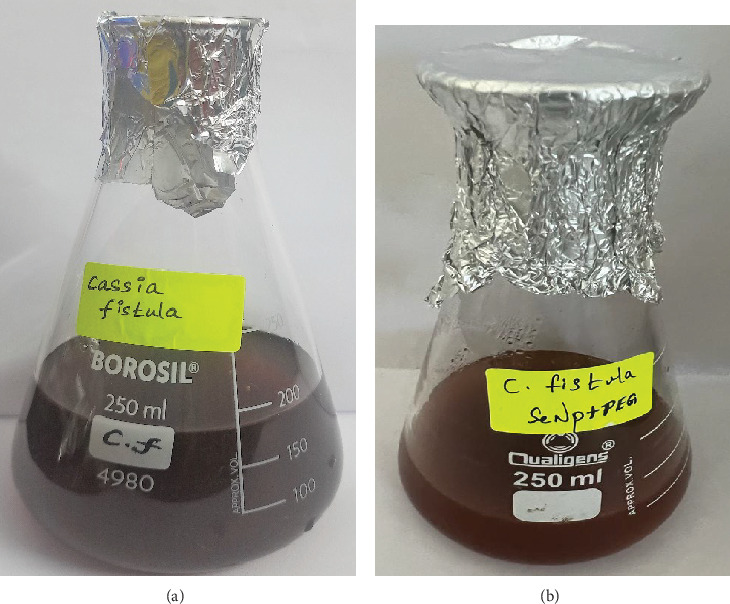
(a) *Cassia fistula* flower (CFF) extract. (b) Fabricated CFF-SeNC.

Sodium selenite was mixed with CFF extract (Figure 1a), which aided in reducing selenite salt to zero-valent Se (Se^0^). The appearance of red confirmed this; adding a PEG to the synthesised CFF-SeNP resulted in the formation of CFF-SeNC (Figure 1b). Further characteristic studies confirmed the coating of PEG.

Sodium selenite was reduced to Se^0^ by CFF extract, forming red-coloured CFF-SeNP, and PEG coating led to CFF-SeNC formation.

### 3.2. Characteristic Studies of CFF-SeNC

#### 3.2.1. UV-VIS Spectral Analysis

**Figure 2:**
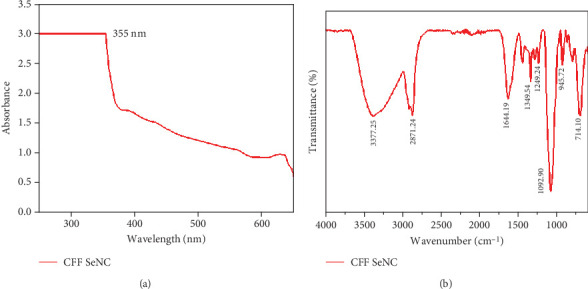
(a) UV-visible spectral analysis of CFF-SeNC. (b) FTIR spectrum of CFF-SeNC.

The UV-visible profile of CFF-SeNC depicted a peak with absorption maxima at 355 nm, which shows that the phytochemical content of CFF extract has involved the formation of Se by reducing sodium selenite (Figure 2a).

#### 3.2.2. FTIR Spectroscopy

FTIR peaks of CFF-SeNC were at 3377.25 cm^−1^ representing O–H stretch of phenols, 2871.24 cm^−1^ denotes to C–H stretch [24], 1644.19 cm^−1^ and 1349.54 belonging to carbonyl functional group and CH_2_ symmetric stretch [25], 1249.24 cm^−1^ corresponding to C–N stretching amines, 1092.90 and 945.72 cm^−1^ represent C–O stretching [26] and glycosidic linkage [25], 714.10 cm^−1^ refers to bending of C–H group. The shifts in the vibrational peaks confirm that the phytochemicals of CFFs are involved in forming nanocomposites (Figure 2b).

The UV-VIS peak at 355 nm confirms the reduction of sodium selenite by CFF phytochemicals in CFF-SeNC synthesis. FTIR analysis confirmed the characteristic functional group vibrations present in CFF-SeNC.

#### 3.2.3. SEM-EDX

**Figure 3:**
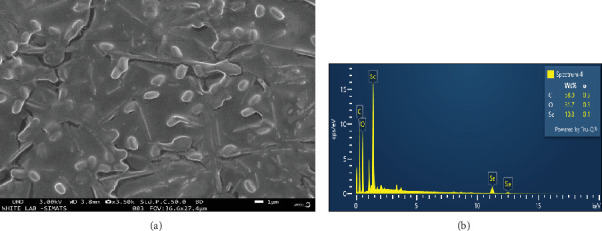
(a) SEM image of CFF-SeNC. (b) EDX spectra of CFF-SeNC.

The structural surface of CFF-SeNC was studied by SEM (Figure 3a), which revealed predominantly polymeric oval-shaped morphology with few needle-like structures, which also looks like the outer matrix covering the core-shell, rough surface with less agglomeration and size measuring in the average range of diameter 702.62 nm. The EDX spectra of CFF-SeNC (Figure 3b) have intensity peaks at 1.37 and 11.2 keV, confirming the Se content in the synthesised nanocomposite. The weight percentage of elements found in CFF-SeNC was 58.0% of carbon, 31.7% of oxygen, and 10.3% of Se. The low sigma value (*σ*) denotes the uniform distribution of the element in the sample, which is 0.1 for Se and that of C and O is 0.3, thus showing that carbon and oxygen are distributed unevenly in the synthesised nanocomposite.

SEM-EDX analysis of CFF-SeNC revealed an oval-shaped polymeric morphology with core-shell features, confirmed Se presence, and showed uniform Se distribution.

#### 3.2.4. XRD Spectrum

**Figure 4:**
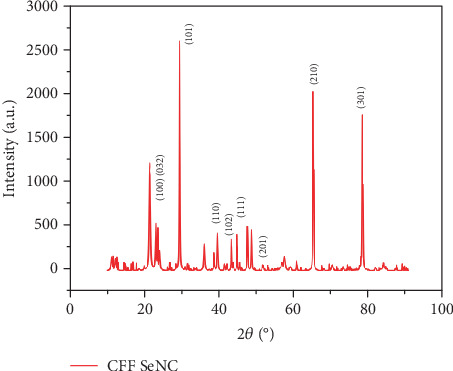
XRD peak of CFF-SeNC.

The crystalline nature of the synthesised CFF-SeNC was depicted by XRD analysis (Figure 4). The sharper peaks of XRD revealed that the synthesised CFF-SeNC is of crystalline form. The XRD results showing (JCPDS File No. 06-0362) degrees of diffraction peaks with lattice planes at 23.45° (1 0 0), 29.30° (1 0 1), 39.34° (1 1 0), 43.01° (1 0 2), 44.55° (1 1 1), 51.59° (2 0 1), 64.95° (2 1 0), and 78.09° (3 0 1) confirm the presence of Se, and the peak at 23.45° additionally corresponds to PEG with lattice plane (0 3 2) further affirming the PEG present in the synthesised CFF-SeNC.

XRD analysis confirmed the crystalline nature of CFF-SeNC with characteristic Se peaks and a distinct PEG-related peak at 23.45°, validating successful PEG incorporation.

### 3.3. Antimicrobial Efficacy

**Figure 5:**
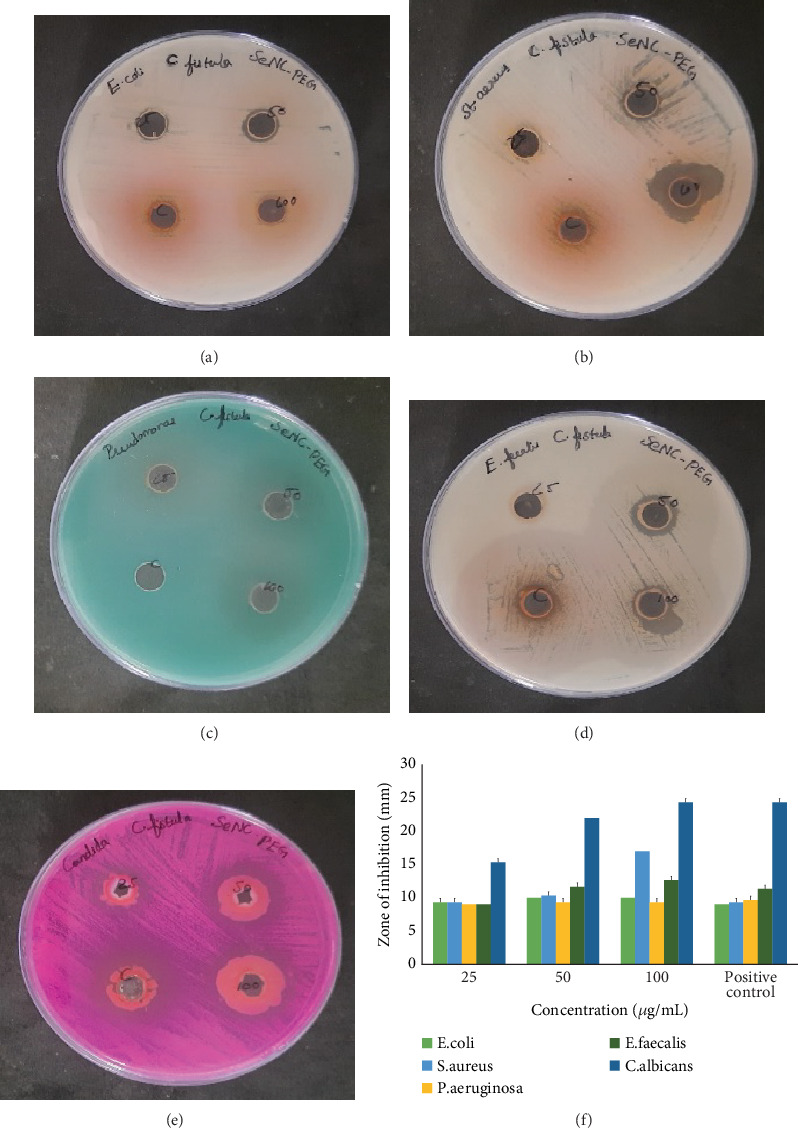
Antimicrobial efficacy of CFF-SeNC. (a) *Escherichia coli*. (b) *Staphylococcus aureus*. (c) *Pseudomonas aeruginosa*. (d) *Enterococcus faecalis*. (e) *Candida albicans*. (f) Graphical representation of antimicrobial efficacy. The experiment was performed in triplicate (*n* = 3), and the results are represented as mean ± standard deviation.

The efficacy of CFF-SeNC against pathogens was studied using a well diffusion method. The synthesised nanocomposite possessed no significant activity for *E. coli* and *P. aeruginosa*. The inhibition zone for *E. faecalis* at 100 *μ*g/mL was 12.67 ± 0.58 mm, and at the same concentration, *S. aureus* exhibited activity with a zone size of 17.00 ± 0.00 mm. However, the CFF-SeNC exhibited better activity against *C. albicans* with zone diameters 15.33 ± 0.58, 22.00 ± 0.00, 24.33 ± 0.58, and 24.33 ± 0.58 mm for 25, 50, and 100 *μ*g/mL and standard (25 *μ*g/mL of SeNP) (Figures 5a, 5b, 5c, 5d, 5e, and 5f).

CFF-SeNC showed notable antimicrobial activity against *S. aureus* and *E. faecalis* and strong dose-dependent antifungal activity against *C. albicans* but was ineffective against *E. coli* and *P. aeruginosa*.

### 3.4. Evaluation of Antioxidant Potency

#### 3.4.1. DPPH Radical Scavenging Assay

**Figure 6:**
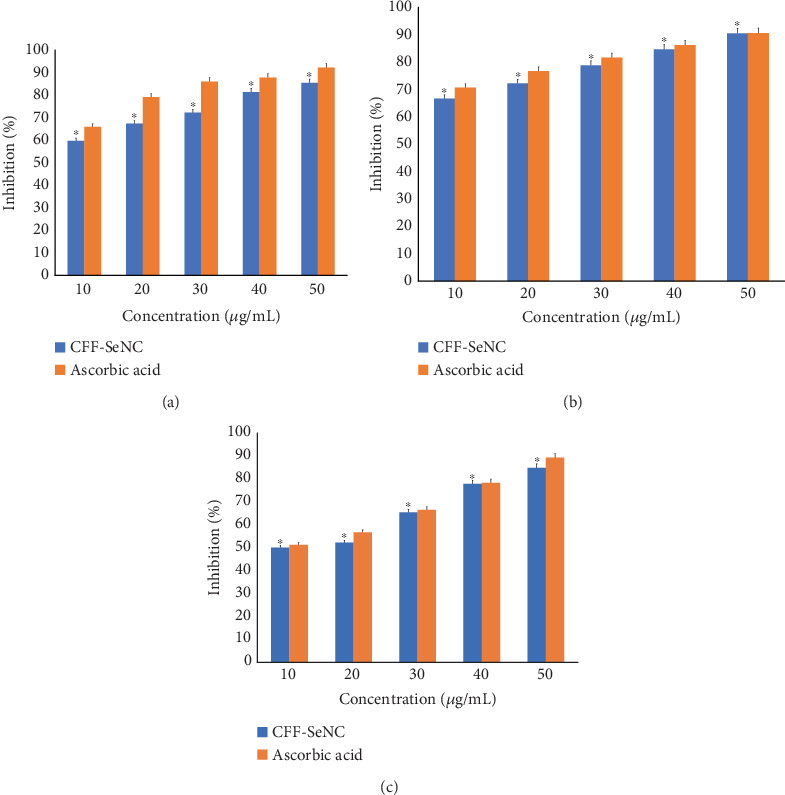
Antioxidative potency of CFF-SeNC with ascorbic acid (standard). (a) DPPH activity. (b) ABTS assay. (c) H_2_O_2_ scavenging assay. The experiment was performed in triplicate (*n* = 3), and the results are represented as mean ± standard deviation.

The free radical sequestration capacity of CFF-SeNC by DPPH assay revealed that the inhibiting percentage at 50 and 10 *μ*g/mL was 85.51% ± 0.79% and 59.77% ± 0.76%. Ascorbic acid (standard) showed 92.24% ± 0.79% and 65.97% ± 1.36% at 50 and 10 *μ*g/mL (Figure 6a).

#### 3.4.2. ABTS Assay

The capacity to sequester free radicals through ABTS cation scavenging assay showed that the sample (CFF-SeNC) and standard (ascorbic acid) inhibition percent at 50 *μ*g/mL was 90.53% ± 0.90% and 90.62% ± 0.85% and at 10 *μ*g/mL was 66.67% ± 0.79% and 70.66% ± 0.81%, respectively. The results show that the sample's antioxidative capacity is almost equal to that of the standard (Figure 6b).

#### 3.4.3. Hydrogen Peroxide Scavenging Assay

The results of the H_2_O_2_ assay exhibited the sequestration ability of CFF-SeNC and ascorbic acid at 50 and 10 *μ*g/mL, which were found to be 84.87% ± 0.80% and 89.25% ± 0.61% and 50.05% ± 0.95% and 51.24% ± 0.70%, respectively. The results of this assay reveal that the ability of the sample to scavenge the highly reactive free radicals is equally efficient to the standard used in the study (Figure 6c).

### 3.5. Anti-Inflammation Studies

#### 3.5.1. Egg Albumin Denaturation Assay

**Figure 7:**
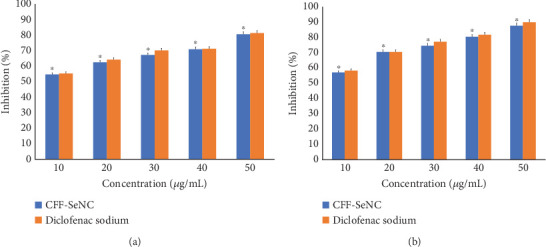
Anti-inflammatory efficiency of CFF-SeNC with standard (diclofenac sodium). (a) Egg albumin denaturation assay. (b) HRBC assay. The experiment was performed in triplicate (*n* = 3), and the results are represented as mean ± standard deviation.

Sample anti-inflammation efficiency was studied using egg albumin denaturation compared to the positive control (diclofenac sodium). The anti-inflammatory efficiency of CFF-SeNC at 50 and 10 *μ*g/mL was 80.61% ± 0.87% and 54.66% ± 0.98%, respectively. The same for diclofenac sodium was measured as 81.33% ± 0.95% and 55.38% ± 0.94% at 50 and 10 *μ*g/mL (Figure 7a).

#### 3.5.2. HRBC Membrane Stabilisation Assay

Upon analysing HRBC membrane stabilisation, the anti-inflammation ability of CFF-SeNC and diclofenac sodium was known to be 57.10% ± 0.83% and 58.33% ± 0.77% at 10 and 50 *μ*g/mL; the activity percent was 87.78% ± 0.88% and 90.05% ± 1.00%, respectively (Figure 7b).

The above results of antioxidative and anti-inflammatory analysis prove that the inhibiting percentage elevates along with the increasing concentration, thus revealing the direct proportionality of concentration to their activity.

CFF-SeNC demonstrated strong antioxidant activity in DPPH, ABTS, and hydrogen peroxide scavenging assays, showing comparable free radical inhibition to ascorbic acid at both 10 and 50 *μ*g/mL concentrations, with the highest scavenging potential through the ABTS assay.

CFF-SeNC exhibited significant anti-inflammatory activity in both egg albumin denaturation and HRBC membrane stabilisation assays, with efficiency comparable to diclofenac sodium at both 10 and 50 *μ*g/mL concentrations.

### 3.6. Biocompatible Property: Brine Shrimp Lethality Assay

**Figure 8:**
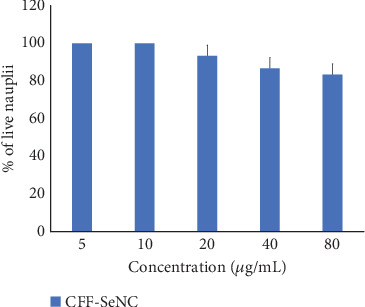
Biocompatibility of CFF-SeNC through brine shrimp nauplius lethality assay during 48 h of treatment. The experiment was performed in triplicate (*n* = 3), and the results are represented as mean ± standard deviation.

The biocompatible capability of CFF-SeNC was assessed by performing an assay on brine shrimp nauplii's lethality. The samples had no lethal effect, showing 100% viability at the end of 24 h of treatment. On further observation at 48 h, the percentage of viability decreased to 83.33% ± 5.77%, 86.66% ± 5.77%, and 93.33% ± 5.77% for synthesised CFF-SeNC at concentrations of 80, 40, and 20 *μ*g/mL while the minimal concentrations of 5 and 10 *μ*g/mL possessed no lethality. In the well without a sample (control), all the nauplii were found alive (Figure 8).

CFF-SeNC showed high biocompatibility in the brine shrimp lethality assay, with no toxicity at lower concentrations and over 80% viability even at higher doses after 48 h.

### 3.7. In Vitro Cell Line Studies

#### 3.7.1. Cytotoxicity and Anticancer Efficacy

**Figure 9:**
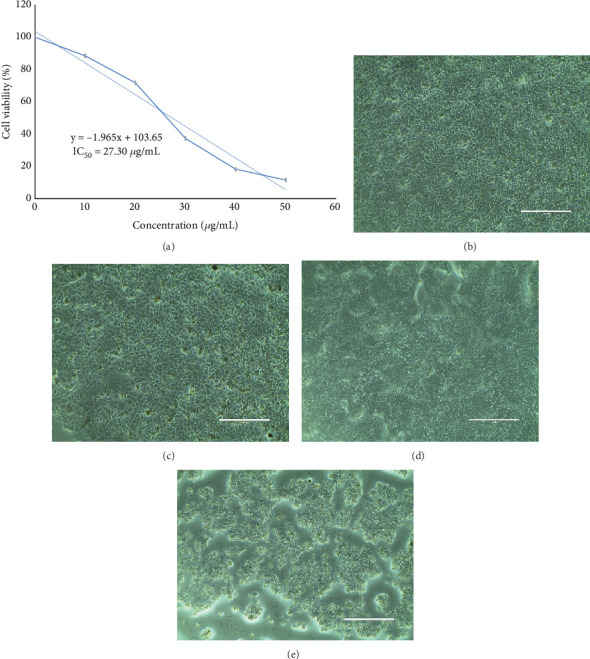
Morphological alterations in Vero and HepG2 cells by MTT assay. (a) Graph depicting IC_50_ dose of CFF-SeNC on HepG2 cells. (b) Vero cell negative control. (c) Vero cells treated with CFF-SeNC. (d) HepG2 cell negative control. (e) HepG2 cells treated with CFF-SeNC. The experiment was performed in triplicate (*n* = 3), and the results are represented as mean ± standard deviation.

Treatment on *Vero* cells to measure the cytotoxicity was quantified by MTT assay for CFF-SeNC exhibited 89.08% ± 0.72% viable cells at 50 *μ*g/mL. At the same dosage, the anticancerous efficiency achieved for CFF-SeNC was 11.47% ± 0.90% viability on HepG2 cells; thus, the obtained results depict the potency of the sample towards cancer cells, even though the lower concentration (10 *μ*g/mL) had viable cells of 88.44% ± 1.10%. The IC_50_ dose of CFF-SeNC was calculated to be 27.30 *μ*g/mL (Figure 9a).

Upon treatment with the CFF-SeNC, the *Vero* cells (Figure 9b,c) did not show any notable alteration in the morphology, whereas HepG2 cells (Figure 9d,e) lost their contact with each other, got shrunk, and became rounded off, losing their specialised morphological nature. The negative control well, which was treated with molecular grade water, has retained its structure.

CFF-SeNC showed strong anticancer activity with an IC_50_ of 27.30 *μ*g/mL, significantly reducing HepG2 cell viability and altering their morphology while exhibiting minimal cytotoxicity and no morphological changes in *Vero* cells.

#### 3.7.2. Dual Staining: AO/PI Apoptotic Study

**Figure 10:**
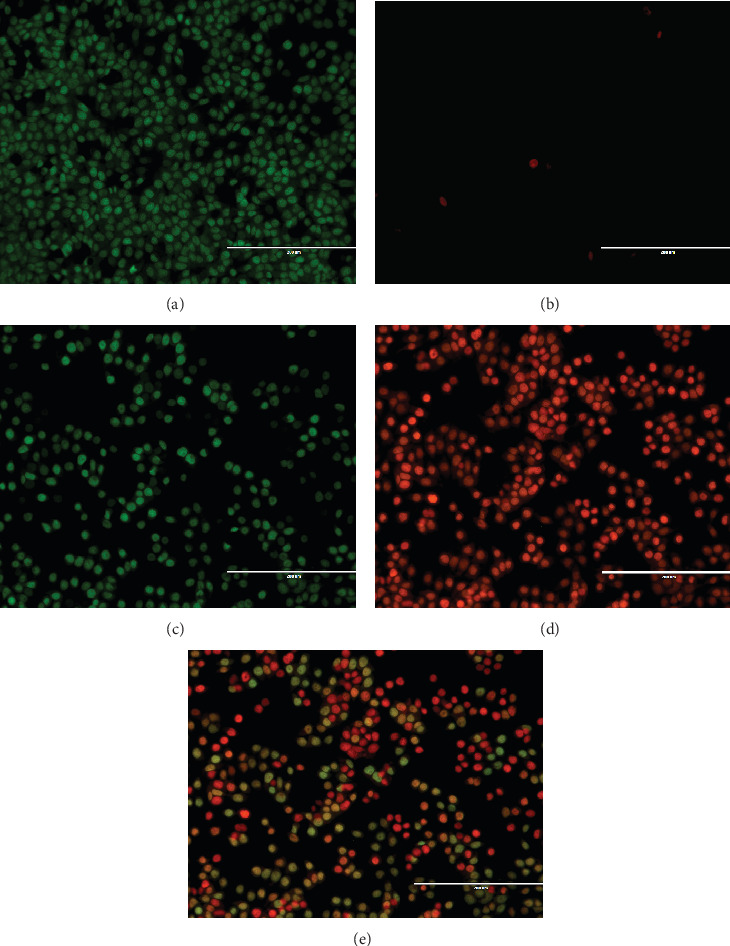
AO/PI staining on HepG2 cells. (a) AO-stained control. (b) PI-stained control. (c) AO-stained treated with CFF-SeNC. (d) PI-stained treated with CFF-SeNC. (e) AO/PI-stained treated with CFF-SeNC.

The dual-stained HepG2 cells treated with the IC_50_ concentration of the sample were viewed through fluorescent microscopy. The cells showed fluorescence in green, denoting their survival (intact) and early apoptotic, orange, and red colours, denoting late apoptotic and necrotic cells, respectively. Almost all the cells in the control well were showing green colour fluorescence, proving the cells' survival. In CFF-SeNC–treated well, the green coloured cells (live cells) are much lower compared to the number of orange and red fluorescence cells (late apoptotic and necrotic cells). The obtained results indicate that the CFF-SeNC is more potent against HepG2 cells. The higher effectiveness of CFF-SeNC might be attributed to its better permeability due to the presence of PEG (Figures 10a, 10b, 10c, 10d, and 10e).

Fluorescence microscopy revealed late apoptotic and necrotic cells on treatment with CFF-SeNC at IC_50_ concentration, which might be due to enhanced cellular permeability from PEG coating.

#### 3.7.3. Cell Cycle Analysis

**Figure 11:**
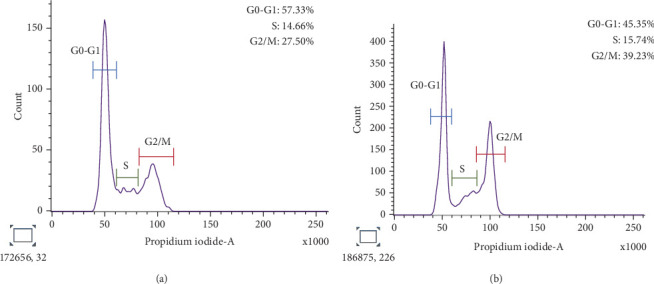
Cell cycle arrest graph on HepG2 cells. (a) Control. (b) Treated with CFF-SeNC.

Flow cytometric analysis of HepG2 cells treated with CFF-SeNC to study the cell cycle phases compared to untreated cells showed a decrease in the percentage of cells from 57.33% to 45.35% in the G0–G1 phase. In comparison, there was a slight increase in HepG2 cells from 14.66% in untreated to 15.74% in treated during the S phase. The cell cycle arrest was at the G2/M phase, with the percentage of apoptotic cells increasing to 39.23% for CFF-SeNC–treated HepG2 compared to the control with 27.50% of apoptotic cells (Figure 11a,b).

Flow cytometry showed CFF-SeNC–induced cell cycle arrest at the G2/M phase in HepG2 cells, increasing apoptosis from 27.5% to 39.23% compared to untreated cells.

#### 3.7.4. Annexin V/PI Staining Studies

**Figure 12:**
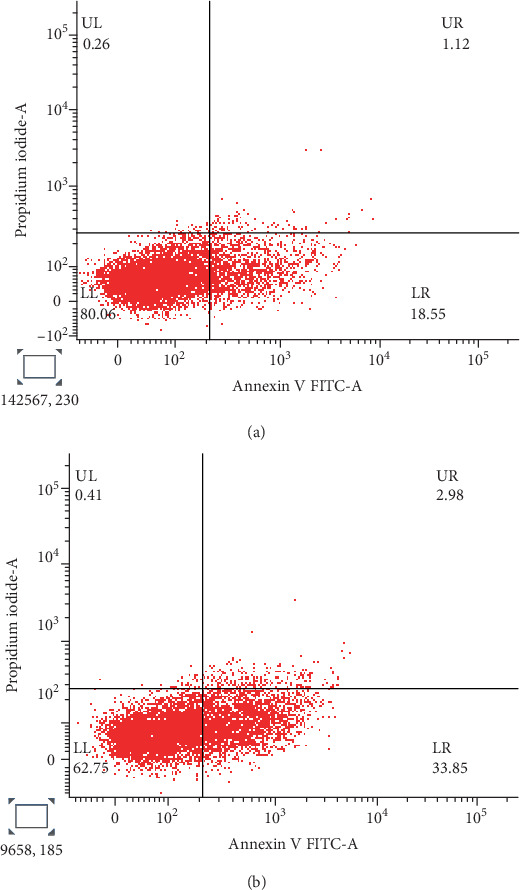
Annexin V/PI staining on HepG2 cells. (a) Control. (b) Treated with CFF-SeNC.

The apoptotic stages of CFF-SeNC–treated HepG2 cells studied using Annexin V/PI depicted that 33.85% of cells were at early apoptosis compared to 18.55% in untreated HepG2 cells. The percentage of live cells decreased to 62.75% in treated cells, which was 80.06% in untreated cells. In addition, treated cells had 2.98% in late apoptosis and 0.41% in the necrotic stage. This clearly proves that the sample CFF-SeNC has effectively induced cell death in treated HepG2 cells (Figure 12a,b).

Annexin V/PI staining showed that CFF-SeNC treatment significantly increased early apoptosis and reduced live HepG2 cells, confirming its effective induction of cell death.

## 4. Discussion

Therapeutical herbs have prevailed more from the olden days for enhancing the human immune system and treating many chronic ailments. Across the globe, the death rate resulting from cancer is in millions being increased each and every year, which has led many researchers to study the improving ability of plant-based products in treating cancer patients [27]. Nanomaterials fabricated utilising plants, flowers, barks, seeds, or any parts of plants have been gaining interest in the medicinal field, targeted delivery of therapy agents. Biological polymers have the advantages of sustainability, compatibility, and enhanced efficacy; thus, encapsulating the NPs or nanodrugs has gained importance in treatments [28]. A drug named Pegasys is an interferon, a drug to treat hepatitis B and C viruses (HBV and HCV) that has been linked to PEG, which enhanced its shelf-life [29]. Another study on MCF-7 cell lines proved that the efficiency of SeNPs has a significant enhancement in inhibiting its growth when coupled with polymers poly(lactic-co-glycolic acid–poly(ethylene glycol)-folic acid [30].

The main aim of the current research work is to evaluate the anticancer efficiency of CFFs mediated by SeNPs encapsulated using PEG. The phytoconstituents of *Cassia* flowers have aided in reducing the sodium selenite to Se ions [31]. The synthesised CFF-SeNC was characterised, and the UV-VIS analysis showed a maximal peak at 355 nm. This is in accordance with the previous work involving *Aloe vera* extraction for designing SeNP, which depicts the highest absorption peak at 350 nm [32].

The role of functional groups present in the flowers of *C. fistula* to stabilise and reduce selenite salt to Se^0^ was further confirmed by FTIR. The phytoconstituents of CFF, namely, phenols, amines, carbonyls, and glycoside bonds of PEG, have acted as capping agents and catalysed reduction during the fabrication process, which also indicates the presence of PEG on the surface of CFF-SeNC [24–26]. The SEM image displayed less agglomeration and an oval shape of the synthesised Se nanocomposite, which is similar to the previous study where the magnetic NPs are more dispersive after the addition of PEG compared to PEG-free MNPs [10]. The peaks of energy dispersive analysis were at 1.37 and 11.2 keV, which were in agreement with the existence of Se ions in CFF-SeNC [33].

The XRD spectrum has sharp peaks, indicating that the synthesised CFF-SeNC is in crystalline form. The study synthesising nano-Se using *Terminalia arjuna* extract has shown that nano-Se has a crystalline as well as amorphous nature [34]. The results of CFF-SeNC contain an XRD peak at 23.45°, which corresponds to the XRD pattern of PEG [35].

There is a predominant increase in pathogens possessing resistance to antibiotics, leading to prolonged infections. The antimicrobial ability of CFF-SeNC against pathogenic infections has shown better control for *C. albicans*. However, CFF-SeNC has not been found much effective against the bacterial strains taken into the study. There was slight activity for *S. aureus* at 100 *μ*g/mL. The study of nanosized Se synthesised from the bacteria *Halomonas elongata* against a fungal strain *C. albicans* proved its effective inhibition in the growth of the organism [36].

Se nanomaterials possess strong antioxidative properties, attributed to their efficiency in balancing the redox potential by scavenging the excess free radicals [37]. The biopotential study through DPPH, ABTS, and hydrogen peroxide activity proved the antioxidative property of CFF-SeNC [32, 38]. The anti-inflammation potency of dextrin-coupled nano-Se has been studied by oral dosage to arthritis-induced rats, proving that these SeNPs possess anti-inflammation efficacy at 250 *μ*g per kilogram of body weight [39]. In another research using wounded rats, the hydrogel formulated in a nanocomposite form of Se linked to bacterial cellulose and gelatin showed efficiency in inhibiting inflammation, thereby helping in wound repair [40]. The egg albumin and membrane stabilisation of human RBC assays have shown that the CFF-SeNC has a better capability to reduce the inflammation. The CFF-SeNC was found to be biocompatible through assessment of lethality using brine shrimp nauplii. The earlier study on brine shrimp lethality suggests that the SeNPs are nontoxic up to a certain concentration. So, the biocompatible nature of Se nanomaterials depends on the dose of material being used for the medical applications [41].

In vitro anticancerous studies of CFF-SeNC have proved to be effective in causing apoptosis in hepatocellular carcinoma (HepG2 cells) without affecting normal cells (*Vero*). Further, the detailed analysis using fluorescence stain (AO/PI), cell cycle arrest, and flow cytometric study by Annexin V/PI on HepG2 has affirmed the stages of apoptosis. CFF-SeNC exhibits enhanced effectiveness in inhibiting the proliferation rate of hepatocellular carcinoma by arresting cancer cell multiplication at the G2/M phase. The polysaccharide-based SeNP study on lung cancer cell lines has shown arresting cells at the G2/M phase [42]. A study carried out using SeNPs designed by utilisation of glucose as a reducing agent has revealed the anticarcinogenic capability and initiation of apoptotic pathways against cancer cells specifically [43]. Previous research showed that SeNPs effectively killed human glioblastoma and melanoma cells [44]. The results of an earlier study on HepG2 cell death by MTT assay revealed that SeNPs have greater potential in lowering the growth rate compared to the drug and drug-conjugated Se [45]. The anticancerous effect of PEG-encapsulated crocin-SeNPs on the A549 cell line was evaluated, which showed enhanced anticancer potency through apoptosis and also has the advantage of pH-assisted release of the drug, which further enhanced the efficacy in treating cancer [46].

The intervention from the results of the present research conveys that the PEG-encapsulated Se nanocomposites fabricated from CFFs were significantly efficient, sustainable, and compatible with pharmacological applications.

## 5. Conclusion

The noteworthy property of naturally available plant-based medicines as a remedy for chronic ailments, with sustainability and biocompatibility in combination with nanotechnology, has a tremendous impact on medicinal research. In our research, we assessed the biomedical effectiveness, such as the antioxidation and anti-inflammatory effects of PEG-encapsulated Se nanocomposite designed from CFFs. The anticancerous studies have affirmed the capability of CFF-SeNC in inhibiting the proliferation rate of hepatocellular carcinoma. The CFF-SeNC has led to an increase in early apoptosis and cell cycle arrest at the G2/M phase in HepG2 cells. Overall, our research findings deliver an enhancement in the therapeutic efficacy of CFF-SeNC as an efficient anticancerous drug, thus making it a better alternative for cancer treatment.

## Data Availability

Data will be made available upon request to the corresponding authors.
